# Barriers and facilitators to the transplant process among patients living with polycystic kidney disease: a qualitative Approach

**DOI:** 10.1186/s12882-023-03174-6

**Published:** 2023-05-01

**Authors:** Juliana Smith, Orlando O. Harris, Deborah Adey, Meyeon Park

**Affiliations:** 1 University of California, San Francisco School of Medicine, 505 Parnassus Ave, San Francisco, CA 94143 United States; 2 Department of Community Health Systems, School of Nursing, University of California, San Francisco 2 Koret Way, N 531E, Box 0608, San Francisco, CA 94143 United States; 3 Department of Medicine, Division of Nephrology, University of California, San Francisco, 400 Parnassus, ACC Box 701 KTU, Box 0532, San Francisco, CA 94143 United States; 4 500 Parnassus Ave MUW 418, Box 0532, San Francisco, CA 94143 United States

**Keywords:** PKD, Polycystic kidney disease, ADPKD, Preemptive transplant, Kidney transplant, Transplant patient perspective

## Abstract

**Background:**

Kidney transplant is the gold standard for renal replacement therapy in patients with autosomal dominant polycystic kidney disease (ADPKD), which is the fourth leading cause of kidney failure. Despite the medical and economic benefits of preemptive kidney transplant over dialysis before transplant, only 9–21% of qualifying patients receive preemptive transplants. Given the low rates of preemptive transplant, the aim of this study was to determine perceived facilitators and barriers to preemptive transplant among ADPKD patients using a qualitative approach.

**Methods:**

Data were collected between July 2021 and January 2022 from virtual individual semi-structured interviews of 16 adult participants with ADPKD. Qualitative analysis of the recorded interviews was conducted to generate themes.

**Results:**

Our findings revealed two themes specific for facilitators to preemptive transplant (social support and patient agency) and three themes specific to barriers for preemptive transplant (inadequate social support, gaps in knowledge, and institutional and systemic policies). The results also include various subthemes and the application of these themes to the social ecological model.

**Conclusions:**

These findings suggest that increasing social support and patient agency, such as through patient navigator programs and encouraging effective communication between health care providers and patients, can facilitate the transplant process. Increasing dissemination of transplant knowledge from institutions and systems to patients through paired kidney exchange education and live donor outreach can also increase timely access to preemptive kidney transplants for patients with ADPKD. Our findings are limited by our single site study in the US, which may not apply to individuals experiencing different social, cultural, and health access conditions.

**Supplementary Information:**

The online version contains supplementary material available at 10.1186/s12882-023-03174-6.

## Background

Autosomal dominant polycystic kidney disease (ADPKD) affects 12.5 million people globally and is the fourth leading cause of kidney failure and renal replacement therapy [1]. ADPKD is characterized by bilateral renal cysts leading to progressive kidney enlargement, abdominal pain and hematuria requiring transplant in 50% of cases [1]. In addition to disease progression and complications affecting quality of life, total incremental costs associated with ADPKD in the United States were estimated to be from $7.6 to $9.6 billion, ranging from $51,970 to $68,091 per individual, in 2018 [2]. About $5.7 billion of these costs came from direct healthcare costs, of which 50% were incurred by individuals with kidney failure [2]. Indirect costs were estimated to be $1.4 billion of the total cost of which unemployment costs and reduced productivity at work were the largest contributors [2].

Kidney transplant, which can be done pre-emptively (in the absence of dialysis), or after dialysis initiation, is the gold standard for renal replacement therapy [3, 4]. In particular, preemptive kidney transplant has been shown to have many advantages, including fewer pre-transplant blood transfusions, and improved long term graft survival for patients, likely due to avoidance of comorbidities from uremia and dialysis, or from improved patient selection [5]. In addition, preemptive transplant has economic benefits like decreased healthcare expenditures on dialysis costs, and opportunity for successful recipients to return to work or enjoy other activities of daily living [5]. For example, one study found that patients with stage 5 chronic kidney disease (CKD) and patients on dialysis had greater six-month direct medical costs compared to post-transplant patients and patients in earlier CKD stages [6]. Despite these benefits, only 9–21% of qualifying patients receive preemptive transplants [7]. Multiple barriers to preemptive transplant may include a lack of an efficient transplant infrastructure, a lack of patient education on live donations, especially paired kidney exchange, a lack of willing, suitable donors, organ allocation policies, late referral to transplant centers and financial concerns for recipients [5, 8]. (Paired kidney exchange allows a recipient to receive a kidney from another recipient’s live donor rather than his/her own donor, due to better compatibility, and is a common practice at our center through participation in the National Kidney Registry.)

Previous studies explored patient perceived barriers to the transplant process using survey questionnaires. For example, using a non-validated questionnaire, Knight et al. (2015) surveyed prospective kidney transplant recipients on factors that hindered or favored transplantation referral before dialysis. They found that ADPKD diagnosis, White recipient racial identity, referral by a transplant nephrologist, education around the option for preemptive transplant, and employment status were associated with referral to preemptive transplant [9]. Similarly, Helmick et al. (2018) used a telephone administered questionnaire to survey living donor kidney transplant recipients, who had either undergone preemptive transplant or not. They found that having ADPKD, longer median time between diagnosis and transplant, and longer time since education about transplant correlated with preemptive transplant [7]. In addition, they discussed the possibility that ADPKD in particular may be associated with another factor not covered by their survey, which may be related to knowledge of transplant experience from other family members previously diagnosed with ADPKD, or early diagnosis allowing for more lead time in the transplant process [7]. In spite of higher rates of preemptive transplant among ADPKD patients, the perception of transplant access and the experience of ADPKD patients facing the transplant process is not well understood.

The experience of patients diagnosed with ADPKD is complex and requires a renewed focus on understanding their lived experiences with navigating the transplant process. Thus, the present study seeks to use a qualitative descriptive approach [10] to elucidate the perspectives of ADPKD patients on the kidney transplant process. Given the uncertainty of factors that may lead to an association between ADPKD and preemptive transplant, the current study also seeks to determine perceived facilitators and barriers to preemptive transplant from the perspective of ADPKD patients accessing care at an academic medical center in San Francisco, California.

## Methods

We chose qualitative description as an appropriate method to describe the phenomena of the lived experiences of patients diagnosed with ADPKD and how they assign meaning and form perspectives related to those experiences [10]. This methodological approach also provides descriptive details about the experiences of ADPKD patients navigation of the transplant process from their perspectives [10]. The study protocol, instruments, and consent forms were approved by the Institutional Review Board of the University of California, San Francisco (UCSF).

### Procedures

Participants were recruited using purposeful sampling techniques [11]. Purposeful sampling was chosen as the preferred sampling framework due to the nature of the topics of interest. Participants were identified via their medical charts available via the University of California, San Francisco’s research database and were recruited if they received care for ADPKD currently or in the past from any of the university’s medical center health systems. We attempted to reduce potential bias by choosing participants across a range of nephrology care providers. Participants were eligible for this study if they were at least 18 years of age, had a diagnosis of ADPKD, and had or were awaiting a kidney transplant. At the end of all study related activities, all participants were offered an honorarium of $25USD for their time.

Starting in July 2021, we first contacted participants via email and then contacted those who did not respond to our initial emails via telephone. The study was conducted in English only. Eligible participants who were interested in the study signed up to be interviewed via several different scheduling platforms such as Doodle, SignUpGenius, or arranged a time outside of the preselected time slots with the lead author via email. Upon selection of a time for their individual interview, participants were emailed their appointment time and a Zoom link to conduct their one-on-one individual interviews. We conducted the interviews via Zoom due to the ongoing COVID-19 pandemic at the time and the restrictions placed on in-person research activities by the university. The lead author obtained written informed consent via DocuSign and also sought verbal consent from all participants before the beginning of the interview. The interviews elicited participants’ perspectives regarding genetic testing, research, and the kidney transplant process, drawing from their interactions with healthcare providers, and navigation of the healthcare system.

### Data collection and analysis

In-depth, individual, semi-structured interviews were the primary source for data collection [10]. Interviews were conducted between July 2021 and January 2022. Based on previous research and clinical experience [10], an in-depth interview guide, consisting of open-ended questions, was developed to cover several specific content areas. This guide also offered consistency throughout the interview process [10]. While the interview guide was developed to provide a systematic organization of the study topic areas, the lead author allowed for flexibility in terms of digressions around less sensitive topics, which provided variations in the interview discussion [12]. All interviews were conducted in English. Our participants were primarily from the San Francisco Bay Area, with some coming from other areas within the state of California. Our sample consisted of (N = 16) participants. All individual interviews were held virtually over video or dial in voice calls via Zoom computer platform. All interviews were conducted by the lead author, a second-year medical student at the University of California, San Francisco, School of Medicine.

Each interview began with a summary of the objectives of the research study to orient and prepare the patient with the topics that would be discussed with them. The lead author conducted each interview, which included warm-up questions to build rapport, followed by various other topics that consisted of open-ended questions about the patient’s experience with being diagnosed with ADPKD, navigating the healthcare system, and transplant (*see* Table 1). The interview ended with participants being asked about their final thoughts around anything they felt was important for the lead author to know that might not have come up during the interview, or any questions they had about the research itself. This was to ensure each participant was given adequate space to express their thoughts honestly and to freely ask questions that were important to them.

**Table 1:** Example Interview Questions by Topic

Topic	Example Questions
**ADPKD Diagnosis Impact**	*Can you tell me what it means to you to be diagnosed with polycystic kidney disease? *How has your life been since you’ve been diagnosed? Has anything changed or remained the same?
**Healthcare Experience**	*How has your experience been as a patient in healthcare? *How has your access been to medical services, such as appointments and tests?
**Transplant Experience**	*What made it easier to receive a transplant? *What have been some challenges in receiving a transplant?
**Conclusion**	*Are there any other things you’d like to tell me, that maybe I didn’t ask you about, but you think are important for me to know?

Qualitative data management and analysis were conducted using the computer qualitative data management and storage software ATLAS.ti (Version 6.2). All in-depth interviews were audio recorded and immediately transcribed verbatim before the start of the analysis process. Each audio file of the recorded interview was de-identified and assigned a six-digit identifier. The recordings were transcribed into text via an audio transcription site and any names mentioned in the interviews were replaced with pseudonyms. The transcriptions were uploaded to the computer software program ATLAS.ti for qualitative data management and storage.

The qualitative analysis process included code development, creation of a codebook, and categorization of codes, which then led to the formation of thematic statements [13]. Code development and extraction involved an iterative process that included consultations with qualitative experts as well as clinicians with expertise with the research topic area [11, 13]. Each line of the transcript was analyzed to allow for codes to emerge from the data (without *a priori* codes). Transcripts were initially analyzed line by line by the first author to identify specific factors related to the kidney transplant process. Additionally, using an open-coding technique, large portions of the text in each transcript were tagged or coded to represent a specific facilitator or barrier to kidney transplant (e.g., family member donor) [14]. The coding of key ideas allowed for organization and assessment of these ideas across multiple participants’ data [13, 15].

A codebook was created at the onset as codes were being generated. Codes were analyzed and compared with each other throughout the process in order to refine or consolidate codes that conveyed similar ideas, which later helped to inform the development of the themes and subthemes. For example, the codes “family member donor” and “altruistic donor” were refined into the code “living donor” to be organized under the transplant facilitators category. The coding process continued until all key ideas in the transcripts were represented. The next step in the analysis process was the clustering of codes together to form categories. For example, the codes (e.g., living donor) were categorized into facilitator themes (e.g., social support) and barrier themes that would later generate thematic statements for each subtheme (e.g., knowing a living donor). Peer review of the codebook, code categorization, and themes with a qualitative analysis expert (O.H.) ensured consistent and valid coding with the best possible code to represent the data [13, 16]. Furthermore, debriefing with members of the writing team allowed concerns and disagreements to be addressed and resolved through consensus. Analysis continued until saturation was achieved; thereby, no new findings emerged from the analysis [15].

The analysis of data was further guided by the social ecological model (SEM), developed by Bronfenbrenner (1977) [17]. The SEM is based on five levels impacting social and health behaviors starting from the individual level, then followed by the interpersonal, community, institutional and system levels. It recognizes that these micro (individual), meso (institutional) and macro (systemic) factors all interact to contribute to one’s health related behaviors and health outcomes. Each theme was organized into one or more levels of the SEM. For example, the facilitator theme of social support was associated with the interpersonal level of the SEM because it is based on relationships the patient has with their family, friends, and health care providers.

## Results

### Sociodemographic characteristics

Table 2 presents the sociodemographic characteristics of the sample. A total of 51 adult individuals were contacted for participation in the present study. Of those contacted for participation, only 16 (31%) agreed to participate. The mean age of the participants was 54.5 years (Range = 32–74 years). A majority of the sample (81%) identified as White American, and 81% had had a pre-emptive kidney transplant. The mean age at the time of transplant was 49.9 (range 25.8–68.7) with mean 5.1 (SD 1.6) years since transplant at the time of the interview.

**Table 2:** Sociodemographic Characteristics of Interviewed Participants

Characteristics	Total (N = 16) N (percentage)
Age (years) (mean, SD)	54.1 (10.9)
Age at transplant (years) (mean, SD)	49.9 (10.8)
Sex, Male	13 (81%)
Preemptive transplant	13 (81%)
Awaiting transplant	3 (19%)
Time since transplant at interview (years) (mean, SD)	5.1 (1.6)
**Race/Ethnicity**	
Non-Hispanic White	11 (69%)
Non-Hispanic Black	3 (19%)
Hispanic/Latino	1 (6%)
Asian/Pacific Islander	1 (6%)

The data reported in the present study highlights two themes specific to the facilitators to preemptive transplant and three themes specific to barriers to preemptive transplant that emerged after a complete analysis of the data. The first facilitator theme is social support, which includes the subthemes (a) knowing a live donor; (b) receiving financial support from the community; (c) connections to the healthcare field; and (d) access to insurance and finances. The second facilitator theme is patient agency, which includes the subthemes (a) knowledge of paired donor exchange; (b) being proactive; and (c) being educated in navigating the healthcare system. The first barrier theme is inadequate social support, which includes the subthemes (a) lack of insurance and finances and (b) difficulty navigating donor evaluation and scheduling. The second barrier theme is gaps in knowledge, which includes the subthemes (a) lacking knowledge of the transplant process and (b) receiving false or insufficient information on transplant from institutions and the media. The third barrier theme is institutional and systemic policies, which includes the subthemes (a) understanding the organ recipient wait list; (b) chance events; and (c) lack of deceased organ donor availability. Lastly, we applied these themes to the SEM. Table 3 presents the themes, subthemes, exemplar quotes and their alignment with the SEM.

**Table 3:** Thematic categories with themes, subthemes, representative quotes, and relevant SEM framework construct

Theme	Subtheme	Quotes	SEM Framework
Social Support	Knowing a live donor who was a match	“I have a very, very, very healthy wife, so we had talked about if she was a match, which fortunately, she was a very good match.”	Interpersonal
	Social and financial support	“I got a lot of cooperation and assistance from our friends and work and the community in general.”	Interpersonal
	Social connections to healthcare field	“We used our medical contacts because my brother-in-law, I have two brother-in-laws who are doctors, one of them that has nephrology as a… So, they gave us a little hint about what was coming so we could ask, is this coming?”	Interpersonal
	Access to insurance and/or financial means	“So, I had excellent insurance, and I never had any issue with any of my payments or getting treatment approved or anything.”	System
Patient Agency	Knowledge of paired exchange donation	“But by the time we got to me actually needing the transplant, paired exchange was a system that was running like a well-oiled machine, and we just dropped into it.”	Individual, Interpersonal
	Being proactive in care	**“**I was always very inquisitive and active in my care. Maybe that helps, in that I was always proactive.”	Individual
	Educated in navigating healthcare system	“When you have a disease like PKD, I don’t think people know it, including my mom, but the only reason I have a general understanding is because of my minimum education in medicine”	Individual, Interpersonal
Inadequate Social Support	Lacking access to insurance/financial means	“When I first got diagnosed, I wasn’t with [insurance] yet. In fact, I was just a cash customer at that time, and I was without health insurance. And this is prior to the [Affordable Care Act], so right away it was very expensive to get the necessary tests done, to find out that I had this.”	System
	Difficulty navigating donor evaluation and scheduling	“Finding a donor, getting through the process of donor evaluation, and just general evaluation. It’s a lot to deal with.”	Interpersonal, Institutional
Gaps in Knowledge	Lacking knowledge of the transplant process	“I don’t feel anybody ever checked in beforehand because I feel… I can’t say it’d have been prevented because I feel like my age… I still feel I had a hard head. But at the same time, I don’t feel that it was explained to me what’s going on and what’s going to happen.”	Individual, Institutional
	False information on kidney transplant in popular culture	“There’s so much mythology, and some of it is meant to just be entertainment…And even in a recent TV show…there was a scene where they’re all in the dialysis room and somebody brings up the illegal harvest of a random kidney and nobody stomps it down and points out that that’s nonsense.”	Institutional, System
	Poor communication / use of medical jargon	“There’s a difference between a person who’s medically trained writing something for the lay person to read than if you had a lay person go and learn it and then write the literature… even though they read the literature, I don’t think it was informative enough”	Institutional, System
Institutional and Systemic Policies	Waiting to be put on organ recipient list	“Although I was under pain and sickness a lot. But yeah. Not to a point where it had to get to… I’m going to say a 2.0 creatinine or something like that to get on the waiting list.”	Institutional, System
	Chance events	“But they also noticed some spots on my lung, just secondary to the CT scan…So we canceled the transplant.”	Individual, Institutional
	Lack of deceased organ donor availability	“I mean just the wait, you know, that three to five years it can be, I guess the average wait…So I would say, I don’t know, that’s not anyone’s fault, that’s just the system”	System

### Facilitator theme 1: social support

On an interpersonal level, social support emerged as a facilitator of the transplant process as participants could rely on social connections for kidney donors, financial support, and healthcare navigation. Knowing a live donor, such as a family member, friend, or another altruistic donor, who turned out to be a match, allowed participants to receive their transplant sooner; as one participant stated, “the fact that…my daughter was a match, obviously I didn’t have to go on a list and wait years and years”. Another participant described the reassurance of knowing early that her brother did not have ADPKD:

…And so that entire time, that 33 years, I knew and was confident that if my brother was a compatible donor, that I would be bringing my own donor, which meant that I didn’t have to participate in the years and years and years of waiting that a traditional person waiting to get a kidney has to do.

Similarly, receiving social and financial support from family, friends and the community made the transplant process operate more smoothly for participants. Some participants shared how their family and friends supported them in their recovery. For example, one participant expressed that her daughter “was able to help around the house,” while another explained that their parents “helped with getting the house ready and stuff, so I’d be ready for the recovery afterwards.” Other participants described community support: “I got a lot of cooperation and assistance from our friends, work, and the community in general”. Another was “shocked” by the amount of support they received from family, friends.

For some participants, having connections in the healthcare field and knowing others who went through the transplant process helped participants navigate their transplant processes. One participant reported having “medical contacts” in their family who would give them “a little hint about what was coming so we could ask, is this coming?” Another participant knew someone who worked for the university hospital medical center who connected them with resources in the medical center and advised them to “start a monitoring process” early when they were first diagnosed with ADPKD at 18. Another participant found comfort in discussing their fears of transplant with family members who have gone through transplant and “just talked to them a little bit about their surgeries.”

On a systemic level, access to health insurance and financial means was also a form of social support that was highlighted in the participants’ narratives. One participant recognized the difference in health care he received compared to his mother: “so, what insurance you have, the type you have, despite what people say, makes a difference. For me, I pretty much got taken care of wherever I went, even when I was in the [Central] Valley.” Another participant shared how having his “excellent insurance” from employment to cover the transplant alleviated his financial worries. He noted “so, I had excellent insurance, and I never had any issue with any of my payments or getting treatment approved or anything. So that was a big relief and is something I never had to worry about.”

Also on an institutional level, appreciation of medical staff was frequently expressed: “I had nothing but great support, great doctors, nurses, staff. Everyone was fantastic.” One patient expressed appreciation for the professional approach of his team: “Everyone I worked with at all levels, the coordinators, the doctors, the surgeons, everybody was just very welcoming, very nice. They’re very straightforward, which I appreciate. It wasn’t like, there was no sugarcoating. They told me exactly what had to be done and what’s going to be done.”

### Facilitator theme 2: patient agency

Narratives on patient agency on an individual and interpersonal level emerged as participants described having knowledge of paired kidney exchange, being educated in navigating the healthcare system, and being proactive in their health care as facilitators of the transplant process. Knowledge of and participation in paired donor exchange allowed some participants, like this individual, to get their transplant without dialysis: “By the time we got to me actually needing the transplant, paired exchange was a system that was running like a well-oiled machine and we just dropped into it.” In addition to knowledge of donor exchange, knowledge of how the healthcare system works made the transplant process easier to navigate. While some participants described knowing family or friends in the medical field, others, like this participant, had medical knowledge themselves. This participant mentioned that “when you have a disease like PKD [polycystic kidney disease], I don’t think people know it, including my mom, but the only reason I have a general understanding is because of my minimum education in medicine.”

Lastly, some participants felt empowered to advocate for their own health by driving the transplant process and used the term “proactive” to describe themselves: “I would say, specific to the university medical center, and even my original treatment doctor, pre-transplant specialist, and my nephrologist, I was always very inquisitive and active in my care. Maybe that helps, in that I was always proactive.” Another participant described their requirement of having a “good fit with a doctor who was not at all bothered or threatened by me being inquisitive and asking why this, not that, being a fully active member of my care and being willing to give feedback about what my experience was” in order to meet “their needs” and did not seek a doctor that “didn’t like that.” While the themes of social support and patient agency arose from participant narratives that described what facilitated their preemptive transplant, the themes of inadequate social support, gaps in knowledge and institutional and systemic policies arose from narratives as barriers to preemptive transplant.

### Barrier theme 1: inadequate social support

In contrast to having social support, inadequate social support emerged from participant narratives as a barrier to preemptive transplant in terms of difficulty navigating donor evaluation and scheduling without support as well as lacking access to financial support. One participant summarized this point clearly, “finding a donor, getting through the process of donor evaluation, and just general evaluation. It’s a lot to deal with.”

On a systemic level, lacking access to insurance or financial means was a factor that impeded the transplant process. One participant described the difficulty of affording their tests earlier in the transplant process before they got insurance. The participant noted that “when I first got diagnosed, I wasn’t with Kaiser yet. In fact, I was just a cash customer at that time, and I was without health insurance. And this is prior to the [Affordable Care Act], so right away it was very expensive to get the necessary tests done, to find out that I had this. And then, I had to find a way to get into the medical insurance system now that I had a preexisting condition”. Another participant recognized that the overall costs of transplant would be a barrier to most people: “my transplant costs, depending on whether we were using wholesale or retail or whatever the rates were, somewhere in a third of a million dollars. And for most people, that’s an unattainable number if they somehow had to pay for that themselves.”

### Barrier theme 2: gaps in knowledge

Gaps in knowledge was another theme that emerged as a barrier to receiving a preemptive transplant. While some participants acknowledged their own lack of knowledge, they also believed that healthcare providers and institutions could do more to fill those gaps to help patients better navigate the transplant process. For example, one participant shared that they did not feel like health providers “checked in beforehand” and although this may not have prevented prolonging their time to transplant, they said “I don’t feel that it was explained to me what’s going on and what’s going to happen”. Additionally, another participant expressed disappointment with the uncertainty behind the next steps in the process, “I remember that they didn’t lay out the entire 10 steps that they wanted us to go through. It was always take this next step. It wasn’t, you need to do these 10 things in this order in this way to get to the finish line…if we knew what all 10 steps were going to be, especially for my brother, we could have started to program those in.”

Participants also expressed receiving false or poorly accessible information on the transplant process as a barrier to transplant. One participant recognized that the media can spread false narratives of transplant. The participant expressed their concern about a show on TV that they thought spread misinformation about the transplant process. The participant offered this narrative to make their point about media misinformation:

…this new show, and it is about a guy getting ready for a kidney transplant. And even in that show, there was a scene where they’re all in the dialysis room and somebody brings up the illegal harvest of a random kidney and nobody stomps it down and points out that that’s nonsense.

Two participants shared how themselves or their family members did not understand the medical jargon from health care providers even though they had a “formal education.” For example, one participant said:

There’s a difference between a person who’s medically trained writing something for the lay person to read than if you had a lay person go and learn it and then write the literature, because I look at my mother and sister, who both have a formal education and they are like, ‘What does this mean? What are they talking about?

Similarly, another participant explained that some providers spoke to them using medical jargon. This left them confused and with unanswered questions. This participant noted:

You have certain doctors, nurses, nursing staff that talk to you like you have a medical degree. And you have others that talk to you, to explain to you in English terms, just to make it easy for you. A lot of times you look at your lab results, you can’t tell this is high, this is low, what’s good, what’s bad…So, you ask them a question, and it takes forever for them to get back to you, or they don’t explain it where you can understand what’s going on.

Some participants offered unprompted potential solutions to remedy the gap in knowledge as a barrier. One participant suggested patient navigators “to help navigate the system because it’s this complicated system… that’s why there are people who may not get timely transplants because they didn’t know that they should have done this, that, and the other thing and all this.” In terms of disseminating accurate information, one participant expressed the importance of educating PKD patients on paired exchange because they’ve met people who aren’t aware of all their options for pursuing a transplant. They expressed:

“It seems like there’s too many people I’m running into who are having a problem because they’re a difficult match in finding a kidney because they’re waiting for a cadaver or trying to round up a matching donor on their own. And paired exchange solves that. All you’ve got to do is come to the table with a willing donor. Don’t matter if they match or not.”

In terms of making information more accessible to different patients, one participant recognized that people have “different learning mediums”, thus, if health care providers “had different ways to articulate their message about whatever procedure it’s going to be, I think that would be helpful for people.” Similarly, another participant suggested that health care providers spend more time explaining to patients the transplant process and checking for their understanding. They suggested, “to me, just kind of taking this slow with somebody, explaining what’s going on, or making sure that the person understands what they’re saying. I think if that’s the case, I think more people would probably do better.”

### Barrier theme 3: institutional and systemic barriers

On an institutional and systemic level, some participants believed that their transplant process was slowed down by policies making them wait until their kidneys failed to be put on the organ recipient list. Participants wondered why they had to wait to be on the transplant list especially since their PKD diagnosis was received much earlier in adolescence or early adulthood years. One participant made this point clear in their narrative, “…it makes absolutely no sense to be reactive versus proactive…with PKD, all the physicians seem like they all agree at least on a few things, which one being it’s a progressive disease that does not have the ability to heal itself, that will eventually result in kidney function loss or failure”. Other participants reported waiting for the creatinine levels to reach a certain number before being put on the list despite becoming progressively sick. One noted, “Although I was under pain and sickness a lot. But yeah. Not to a point where it had to get to…I’m going to say a 2.0 creatinine or something like that to get on the waiting list…I stalled out at about 2.3 and it just stayed that way for a couple of years. And I was really sick, but not sick enough.” Even when matched with a live donor, institutional policy did not allow for preemptive transplant to occur until creatinine and eGFR levels reached the clinical threshold for transplant. One participant expressed that she had to wait for an extensive period of time until her creatinine reached the threshold to facilitate a transplant. She noted, “I had to go through the process twice of matching because we expired a two year process or whatever window it was.”

Participants recognized that having a long wait time for a deceased donor organ for transplant was largely due to lack of donor organ availability from willing live donors or deceased donors. Appealing to live donors through community or national outreach was one suggestion to increase the availability of live donors that emerged from the narratives. One participant suggested:

You go to the DMV and everybody pushing, donate, donate, donate, donate your organs. I feel if they made more information available to individuals, I feel more people would donate. Finding out after you’re going through the process, if you did donate an organ and your organ went out, you jump to the top of the list. I feel that’s good information to put out there for potential donors, so they’re not like, ‘What happened if mine go out? Then I’m just going to pass away.’

Similarly, another participant felt that legislative changes pushing firefighters and police to check for crime victims’ donor status and allowing their organs to be donated before their bodies were used for investigation, could increase the number of organs available from deceased donors. As a firefighter, this participant shared how in his community when a person dies from a crime, they would leave the victim at the crime scene so the police could do their investigation “instead of doing CPR, putting them on a gurney and tak[ing] them to the hospital to harvest anything.”

Ultimately, the barriers of inadequate social support, gaps in knowledge, and institutional and systemic policies contributed to perceived delays to preemptive transplant for participants. Solutions framed around increasing effective communication of healthcare providers and institutions with patients and changing institutional and systemic policies arose from participants’ narratives.

## Discussion

### Implications

There is limited insight into patient-perceived facilitators and barriers to preemptive transplant among patients with ADPKD. Our study attempts to address this gap through in-depth interviews of patients with ADPKD. Our participants valued social support for facilitating preemptive transplant particularly allowing for access to financial resources, live donors, and healthcare navigation. Conversely, participants felt that inadequate social support prolonged the transplant process whether or not they received a preemptive transplant. This is consistent with a survey-based study examining barriers to preemptive transplant which found that while patients who did not know a living donor were similar in both preemptive transplant and non-preemptive transplant groups, the presence of multiple barriers, including social factors, predicted a lower likelihood of receiving a preemptive transplant [7]. In addition, data shows inequities in transplant access especially among ethnic minorities [5, 18] which is consistent with our study where three participants still awaiting transplant also identified as Black.

Some participants who received a preemptive transplant had agency to advocate for their health and to use their healthcare system knowledge to navigate the transplant process. This is consistent with a qualitative study that found that patients believed that being proactive was important to find information that helped their decision-making about kidney-transplant listing [19]. Similarly, other data show that patients receiving preemptive transplants tend to have higher socioeconomic status and education levels [18, 20]. Although patient agency facilitated the transplant process for some, other participants felt that there was a lack of guidance and education on the transplant process and options offered to them, which impeded their transplant. Similarly, one study found their participants had similar concerns about a lack of knowledge and unclear communication about preemptive transplant [19]. Participants in the current study described solutions to these barriers such as having a patient navigator, a volunteer who has gone through the transplant process or a health care provider, who could work with ADPKD patients from diagnosis until transplant as well as education on living donor options like paired kidney exchange. These navigators could facilitate patient communication with other providers on the team as well as guide patients through the steps for donor evaluation and other aspects of the transplant process. Especially with less advantaged groups, education on different living donor options and access to this education could facilitate access to preemptive transplant [5]. Furthermore, patient-friendly resources about the assessment for receiving a transplant and matching with a living donor would help to support patients throughout their illness journey and give them agency to participate in decisions about their health [19]. Notably, official program educational materials including pamphlets and videos are required to be targeted to a fifth to eighth grade reading level, but the gaps in understanding expressed by some participants in our study suggest that the current format for presenting these materials is not sufficient for comprehension. Some comments also belied lack of understanding for the need for psychosocial evaluation, which is a mandated domain for transplant eligibility.

Lastly, participants believed that institutional and systemic policies affected the waiting time for their transplants, preventing some participants from receiving a preemptive transplant. For example, some participants expressed distress for having to wait until their labs reached more critical values for a transplant even as their PKD diagnosis and prognosis were known for years since adolescence or early adulthood. Considerations for PKD-specific transplant considerations such as planned nephrectomy are not routinely incorporated into transplant program policies. Therefore, proactive steps should be taken to ensure patients with PKD can be properly educated and guided to a preemptive transplant [4, 21]. Guidelines for preemptive kidney transplant requirements not based on eGFR in ADPKD patients could be considered, taking into account disease symptoms and need for nephrectomy, but education about the rationale for national transplant program policies is also needed. Some participants also described potential solutions to the long wait time. For example, one participant (a first responder) suggested increasing the availability of deceased donor organs by implementing protocols to rush potential organ donors to the hospital instead of leaving them at the scene of crimes or accidents. Another suggested increasing outreach to potential living donors in their communities and emphasizing the promise of prioritizing donors for future transplants if needed. Similarly, one study outlined various systemic barriers to preemptive living donor transplant and how they could be mitigated with an efficient transplant infrastructure [5]. For example, addressing living donor safety after transplant including ensuring lifelong donor follow up could help increase willing donors [5].

### Strengths and limitations

A major strength of the present study was our qualitative approach with open-ended questions, allowing participants with ADPKD to elaborate on and describe their own perceived facilitators and barriers to transplant without being limited by options given to them on a questionnaire. Participants could also offer potential solutions to pre-emptive transplant barriers which they believed could have helped them or people like them in their own words. Although there is a risk with qualitative interviews that participants would only express socially desirable views, the participants in the current study were able to describe both positive and negative health care experiences, which suggests they felt comfortable discussing sensitive topics. In addition, the open-ended structure of the interviews gave participants space to question and clarify what was being asked of them. At the end of the interview, participants were encouraged to add any additional information that was not directly asked of them but that they felt would be important for health care providers to know.

Our study is limited by a small sample size overall as well as a lack of geographic diversity, having participants from only one institution in San Francisco, as well as a lack of gender and racial diversity, with only 3 women and 3 Black participants. It is acknowledged that patients from the current study’s institution may have different experiences in accessing healthcare and being supported through the transplant process compared to those at other institutions, which influences their health decision making and transplant experiences. In addition, the majority of the current study’s participants identified as White and male and although these participants described various barriers in their transplant processes, previous research shows White race being more positively associated with preemptive kidney transplant than non-White race [7]. Reasons for greater participation in our study by men rather than women are uncertain but can likely be attributed to small sample size alone. Our study did take place during the COVID-19 pandemic when excess caregiving burdens on women may have restricted participation. As previous research found greater inequities for transplant and healthcare access among ethnic minorities [5], and in certain geographic areas as one participant pointed out, participants from various institutions and from different backgrounds may experience different facilitators and barriers to preemptive transplant. Although we reached out to many participants with PKD of various ethnic backgrounds in the UCSF research database, we were only able to interview the subset that responded. Recruiting participants at other institutions could increase the geographic diversity as well as chances for ethnic diversity to more adequately represent the attitudes of the diverse population of patients with PKD. As it stands, our small, single-site study is limited in generalizability.

Another limitation is not collecting the income and education levels of the participants. It is acknowledged that these factors could affect the participant’s access to facilitators, such as social support and agency, as well as influence the participant’s perception of facilitators and barriers. Further research should include this data to enhance the data interpretation. We were also unable to assess factors of genetic testing and family history as themes in this small study, due to low overall sample size.

Lastly, it is acknowledged that participants’ views provide only the patient insight into factors that affected their transplant processes and the solutions to certain issues. While many participants had healthcare experience themselves or medical knowledge from their social connections, complementary insights from health care providers are also needed to contribute to the discussion around optimizing preemptive transplant for patients with PKD.

## Conclusions

This study provided unique insight into patient perceived facilitators and barriers to preemptive kidney transplant. We found that the main facilitators to preemptive transplant were social support from family, friends, and healthcare providers as well as patient agency to ask critical questions and help to move the transplant process along. The main barriers to preemptive transplant were inadequate social support, including financial means, gaps in knowledge including lack of understanding on donor options, and institutional and systemic policies. Health care institutions could consider a patient navigator program to counsel patients about every step of the transplant process. In addition, health care providers including general nephrologists should be encouraged to assess patient’s understanding of transplant program requirements and to communicate clearly with patients without medical jargon. On an institutional and systemic level, paired kidney exchange education and live donor outreach should be shared with potential organ donors and recipients to help them be able to make an informed decision about transplant and to increase live donor availability for preemptive transplants. Better understanding of perceived barriers experienced by patients may help to improve timely access to kidney transplants for patients with ADPKD.

## Electronic supplementary material

Below is the link to the electronic supplementary material.

Additional File 1 : Barriers to ADPKD Genetic Testing & Pre-emptive Transplant Interview Guide

## Data Availability

The datasets generated and analyzed during the current study are not publicly available due to containing confidential patient information but are available from the corresponding author on reasonable request.

## List of Abbreviations

ADPKD: Autosomal Dominant Polycystic Kidney Disease
PKD: Polycystic Kidney Disease
ESKD: End-stage kidney disease / Kidney failure
CKD: Chronic Kidney Disease
UCSF: University of California, San Francisco
SEM: Social Ecological Model
