# Supplementary material for: Barriers and facilitators to the transplant process among patients living with polycystic kidney disease: a qualitative Approach
Source: BMC Nephrol. 2023 May 1;24:119. doi: 10.1186/s12882-023-03174-6 (PMC10150665; doi:10.1186/s12882-023-03174-6)
Supplement: Supplementary file 1 — Additional File 1 : Barriers to ADPKD Genetic Testing & Pre-emptive Transplant Interview Guide [file 12882_2023_3174_MOESM1_ESM.docx]

**Barriers to ADPKD Genetic Testing & Pre-emptive Transplant Interview Guide**

*The intent of this study is to get to know more about the experiences and perspectives of patients diagnosed with autosomal dominant polycystic kidney disease ADPKD about genetic testing and kidney transplant for ADPKD. We would like to know more about your experience in pursuing genetic testing and any factors that shaped your willingness or unwillingness to do genetic testing. Finally, we would like to also know about your experience in receiving a kidney transplant and any factors that made it easier or harder. First, I want to get to know more about you and your story and then we’ll get to the other stuff.*

**A. Warm-up Questions: Getting to know you! (5 minutes)**

This section of the interview is designed to help the participant and interviewer develop rapport and establish a basic level of comfort. The questions within this section focus on the subject’s upbringing (particularly their home environment, experience of family and childhood).

1. *What would you like for me to call you during the interview? What are your pronouns?*
2. *Where did you grow up?*
3. *What was life like in your neighborhood? Where you close to anyone in your neighborhood? Why? What drew you to them? Did you feel safe there?*
4. *When you were growing up, who lived in your household? Where you close to anyone? If yes, who? Did you feel safe there?*

**B. ADPKD Experience (5 minutes)**

This section is to get the participant’s perspective on having ADPKD and how it affects their daily life.

*Can you tell me what it means to you to be diagnosed with polycystic kidney disease?*

1. *How has your life been since you’ve been diagnosed? Has anything changed or remained the same?*

*3. In your everyday life, for example, yesterday, how were you aware of your illness?*

1. *What problems did the illness cause you? How has it impacted your life?*
2. *What problems did the illness cause your family? How has it impacted your family’s life?*
3. *How are you reminded that you are living with polycystic kidney disease?*

**C. Experience in Healthcare/Research (15 minutes)**

This section is to understand the participant’s experiences in healthcare and research and how it may influence their perspective in participating in research and using medical services.

*1. How has your experience been as a patient in healthcare? (For ADPKD or unrelated)*

*a. How have your interactions been with health care providers such as physicians, registered nurses, etc?*

1. *What about your relationships with nurses? Receptionist? What about other members of the health care staff?*
2. *Did they make you feel welcomed or unwelcomed? How so?*
3. *Do you feel heard? Do they take the time to listen to your concerns?*
4. *Do you trust your provider? Why or why not?*

*2. As a person with ADPKD, how has your care been?*

*a. Do you feel your providers are routinely checking in with you before your symptoms worsen or only when they begin to worsen?*

*3. How has your access been to medical services, such as appointments and tests?*

*a. Are you able to make it to your appointments when you have symptoms early on?*

*b. Are there any barriers to accessing medical services?*

1. *How has COVID impacted your ability to access timely health care services?*
2. *Did you provider switch to telemedicine? If so, what was that experience like? If they did not, how else were you able to interact with your provider?*
3. *What are some things that have or would help make these services more accessible?*
4. *What are some things you wish could have been available to make the process to get diagnosed go more smoothly?*

*4. Have you had any experience/s being a participant in research? If so, please describe that/those experiences?*

*a. How have your interactions been with researchers?*

1. *How have those interactions shape your view of research?*
2. *Does the race of the researcher impact your decision to participate in the research?*

*b. What are some factors that make you more willing to participate in research?*

*c. What are some factors that make you less willing to participate in research?*

*5. What are your thoughts about participating in clinical trials for research?*

1. *Do you trust that the researcher will have your best interest at heart? If so, what lead you to believe that?*

*6. How do you feel different groups of people (such as people of color) are represented in research clinical trials?*

*a. Are they represented fairly? If not, how so?*

*b. Does your perspective on clinical trial representation influence your desire to participate in them?*

*c. Are your attitudes or feeling towards participating in clinical research shaped by historical events? (i.e., Tuskegee, etc.)*

**D. Perspective/Experience with Genetic Testing (15 minutes)**

This section is for understanding the participant’s perspective on genetic testing and how this influences the participant’s willingness or skepticism in getting a genetic test. This section will also explore facilitators and barriers to receiving a genetic test for ADPKD based on the participant’s perspective and experience.

1. *Are you familiar with direct-to-consumer sites like* [*ancestry.com*](http://ancestry.com) *where you can learn about your genetic ancestry (what part of the world your ancestors are from)?*
2. *Have you tried any of those sites? If so, what was that like for you?*
   1. *Did you learn anything? What did you learn? What was that experience like? Did you have any reservations about giving your genetic materials (DNA) to a for profit company?*
3. *Whether you tried any of those sites or not, what are your thoughts about giving your genetic information for a test? What about to a for profit company like ancestry.com? What about a hospital or your doctor?*
4. *Do you feel any distrust or skepticism towards genetic testing for yourself and/or others in your community? Why or why not?*
   1. *Have you had or heard of any experiences with health care providers or researchers about genetic testing that influenced your perspective?*
   2. *Have you known anyone who have had experiences with seeking medical treatment based on genetic results? (i.e., seeking a mastectomy based on BRCA testing).*
5. *Do you have any hopes for genetic testing for yourself and/or others in your community? Why or why not?*
   1. *Have you had or heard of any experiences with health care providers or researchers that influenced your perspective?*
   2. *What information or resources would you need in order to consent to genetic testing?*
6. *When you went to your provider, was testing offered to you for ADPKD before transplant? If so, at what stage/when?*
   1. *Did you feel like you got support from your provider to do genetic testing? Why or why not?*
   2. *Were there any barriers or difficulties with getting tested?*
   3. *Were there any things that made it easier to get tested?*

**E. Perspective/Experience with Transplant (15 minutes)**

This section is for understanding the participant’s perspective on receiving a kidney transplant and the social support you received and/or wished you had received. This section will also explore facilitators and barriers to receiving a transplant based on your perspective and experience.

1. *Have you had a kidney transplant? If so, at what stage/when-how long after diagnosis?*
   1. *How did you become aware that you needed a kidney transplant?*
2. *(If no to #1) Are you in need of a kidney transplant? If so, how did you become aware that you needed a kidney transplant?*
3. *If you had a transplant, did you have any social support, such as from family, friends, etc, for a transplant? If so, please describe.*
   1. *If you were to have a transplant what are some of your concerns? What would make it easier to go through the procedure? What would make it harder? What support do you think you would need? From whom?*
4. *If you have had a transplant, were you on dialysis before or were you able to receive a transplant without dialysis before? If yes, what was that experience like? What barriers did you encounter when accessing dialysis services?*
5. *What have been some challenges in receiving a transplant?*
6. *What made it easier to receive a transplant?*
7. *What are some things you wish could have been available to make getting a transplant go more smoothly?*

**F. Final Thoughts (5 minutes)**

This section of the interview is allotted for the participants to vocalize additional topics of importance and interest that were not covered in the proceeding sections of this guide.

*We’re nearing the end of the interview, but before we conclude I would like to hear about anything you feel we missed.*

1. *Are there any other things you’d like to tell me, that maybe I didn't ask you about, but you think are important for me to know?*
2. *Do you have anything that you would like to ask me?*

**G. Conclusion**

*Thank you for your time and candid answers.*
